# Prevention of Postoperative Hernias in Emergency Surgery – PROPHECY Trial

**DOI:** 10.3389/jaws.2025.14765

**Published:** 2025-07-09

**Authors:** Bartosz Molasy, Mateusz Zamkowski, Kryspin Mitura, Maciej Śmietański

**Affiliations:** ^1^ Medical College, Jan Kochanowski University, Kielce, Poland; ^2^ 2nd Department of Radiology, Medical University of Gdansk, Gdańsk, Poland; ^3^ Faculty of Medical and Health Sciences, University of Siedlce, Siedlce, Poland

**Keywords:** postoperative hernia, port-site hernia, parastomal hernia, emergency surgery, burst abdomen

## Abstract

**Background:**

Postoperative hernias are a common and significant late complication following emergency abdominal surgery. Despite numerous preventive strategies, their incidence remains high, leading to morbidity, reoperations, and reduced quality of life. The PROPHECY trial is designed to assess the incidence of postoperative hernias and identify modifiable perioperative risk factors in patients undergoing urgent and emergency abdominal operations.

**Methods:**

This national, prospective, multicentre, observational cohort study will include adult patients undergoing emergency abdominal surgery for specific indications such as acute appendicitis, cholecystitis, incarcerated hernias, gastrointestinal perforation, or bowel obstruction. The study period extends from 1 June 2025 to 31 December 2026, with recruitment completed by the end of 2025 and follow-up completed by the end of 2026. Data on demographics, comorbidities, surgical techniques, postoperative management, and complication rates will be collected. The primary endpoints are incisional hernia, port-site hernia, and parastomal hernia. Secondary endpoints include surgical site infections (SSI) and burst abdomen. Follow-up will be conducted via structured telephone interviews and clinical or imaging confirmation. Based on power calculations for primary and secondary outcomes, the study will include 500 patients, providing adequate statistical strength while maintaining feasibility across participating centres.

**Trial registration:**

ClinicalTrials.gov ID: NCT06815822.

## Introduction

Emergency abdominal surgery is associated with increased rates of early and late postoperative complications, particularly the development of postoperative abdominal wall hernias [1]. This study protocol outlines the rationale and design of the PROPHECY trial, which will investigate this issue prospectively. Midline incisional hernias are among the most common complications after laparotomy and are associated with reduced quality of life, chronic pain, and need for complex reoperations [2, 3]. Despite a large number of analyzes on ways to reduce the incidence of this complication, it is still an important surgical problem [2]. Studies encompassing both elective and emergency procedures report postoperative hernia rates ranging from 2% to 40% [4]. This variation may be attributable to the often asymptomatic nature of certain hernias. An example are port-site hernias although generally perceived as rare, may occur more frequently than previously recognised, especially at umbilical sites [5]. Another significant surgical problem is parastomal hernias, occurring in up to 50% of patients with stomas [6]. The variability in reported incidence is also influenced by the method and duration of follow-up. True incidence rates are likely underreported, particularly in emergency surgery patients, where postoperative follow-up is frequently inconsistent or lacking.

Numerous studies and guidelines have investigated factors influencing hernia formation. Patient-related risk factors include diabetes, obesity, chronic pulmonary disease, immunosuppression, and a history of abdominal surgeries [7]. The guidelines of the European Hernia Society emphasize the need for further research into methods of preventing the occurrence of postoperative hernias [2]. Procedure-related factors such as the type of incision, suture material, fascial closure technique, and use of surgical drains also play a role in hernia pathophysiology [1, 4, 8]. The World Society of Emergency Surgery (WSES) and European Hernia Society (EHS) guidelines recommend the “small bites” technique for fascial closure using slowly absorbable sutures, although most of the supporting evidence is derived from elective surgery [1, 9]. Despite these recommendations, high-quality prospective data focusing exclusively on emergency surgery settings are limited. These guidelines highlight the importance of conducting prospective studies to establish the efficacy of this technique in urgent and emergency surgical procedures [1].

Management of surgical wounds and abdominal wall closure in the emergency setting remains highly variable, with limited evidence guiding best practice. Surgical site infection—affecting approximately one-third of patients undergoing emergency procedures—is also a well-established risk factor for postoperative hernia development [2, 7] A less common but clinically significant complication, which also contributes to hernia formation, is wound dehiscence or evisceration [10]. Therefore, prevention of such early postoperative complications is crucial in reducing the risk of subsequent hernia formation. Furthermore, there is a lack of consensus regarding the effectiveness of postoperative interventions aimed at hernia prevention, such as the use of abdominal binders or recommendations concerning the timing of return to full physical activity [9].

Given the substantial clinical burden posed by postoperative hernias and the paucity of targeted data in emergency surgery, the PROPHECY trial aims to quantify hernia incidence and assess modifiable perioperative risk factors within this high-risk cohort.

## Main and Secondary Hypotheses

### Primary Hypothesis

The incidence of postoperative hernias (incisional, port-site, and parastomal) in patients undergoing emergency abdominal surgery is high and is associated with modifiable perioperative risk factors.

### Secondary Hypotheses

• Specific surgical techniques, including fascial closure using the “small bites” method and slowly absorbable sutures, are associated with a lower incidence of postoperative hernia.
• Postoperative management strategies, such as the use of abdominal binders, NPWT, and early mobilisation, may reduce hernia occurrence.
• Infection of the surgical site (SSI) and burst abdomen significantly increase the risk of postoperative hernia formation.

## Methods and Design

### Study Design and Registration

The PROPHECY trial is a prospective, multicentre, observational cohort study conducted across multiple surgical centres in Poland. The study will run from June 2025 to December 2026, with follow-up and final data analysis planned for early 2027. It is coordinated by the Department of Surgical Sciences and the Laboratory of Medical Genetics of the Medical College, Jan Kochanowski University in Kielce, Poland. Ethical approval was granted by the Institutional Bioethics Committee (Decision No. 5/2025), and the study is registered at ClinicalTrials.gov (NCT06815822). This protocol is published prior to patient enrolment in order to enhance methodological transparency, facilitate reproducibility, and reduce the risk of selective reporting.

### Study Timeline

• Centre enrolment: May 2025
• Patient recruitment: 1 June 2025–31 December 2025
• Follow-up period: 1 January 2026–31 December 2026
• Data analysis: January 2027
• Expected publication: February/March 2027

### Eligibility Criteria

#### Inclusion Criteria

• Age ≥18 years
• Undergoing emergency abdominal surgery for one of the following indications:
  ▪ Acute appendicitis
  ▪ Acute cholecystitis
  ▪ Incarcerated abdominal hernia
  ▪ Gastrointestinal perforation
  ▪ Bowel obstruction

#### Exclusion Criteria

• Patient refusal to participate
• Open abdomen technique utilised intraoperatively

### Data Collection

Clinical and demographic variables to be collected include age, sex, body mass index (BMI), comorbidities, ASA (American Society of Anesthesiologists) classification, and history of prior abdominal surgery. Perioperative variables include the type of surgical approach (open vs. minimally invasive), operative technique, use of drains, irrigation or lavage of the wound, fascial closure method (technique and material), and wound management strategies including NPWT. Postoperative variables such as the use of abdominal binders or elastic taping and time to full physical activity resumption will also be recorded.

### Follow-Up Protocol

All participants will be followed up 12 months after discharge via telephone by a trained physician using a structured interview. In cases of suspected hernia, patients will be invited for outpatient clinical examination and ultrasound or CT imaging to confirm the diagnosis.

### Study Endpoints

#### Primary Endpoints

• Incidence of incisional hernia at the surgical site
• Incidence of port-site hernia following laparoscopy
• Incidence of parastomal hernia following stoma formation

#### Secondary Endpoints

• Occurrence of surgical site infection (SSI)
• Occurrence of burst abdomen

### Statistical Analysis

The primary and secondary outcomes will be expressed as proportions with 95% confidence intervals. Continuous variables will be described using medians and interquartile ranges and analysed using Student’s t-test or the Wilcoxon test as appropriate. Categorical variables will be compared using the Chi-squared test. Univariate and multivariate logistic regression models will be applied to identify independent risk factors for postoperative hernia formation. Relative risk (RR) with 95% CI will be calculated. A p-value <0.05 will be considered statistically significant. All analyses will be conducted using R software in RStudio.

### Sample Size Justification

To estimate a 5% precision for postoperative hernia incidence with 95% CI, a minimum of 255 cases is required. For port-site hernias, at least 188 patients are required; for parastomal hernias, 349. For secondary outcomes, the required sample sizes are 255 for SSI and 93 for burst abdomen. Sample size considerations are summarised in Table 1. To detect a moderate effect size (Cohen’s d = 0.3) with 80% power at α = 0.05, the required sample size is.

• Chi-squared test (1 df): 88 patients
• Chi-squared test (5 df): 143 patients
• Student’s t-test: 102 patients

**TABLE 1:** Sample size rationale.

Endpoint	Sample size	Notes
Incisional Hernia	255	To estimate with 5% precision, 95% CI
Port-site Hernia	188	To estimate with 5% precision, 95% CI
Parastomal Hernia	349	To estimate with 5% precision, 95% CI
Surgical Site Infection (SSI)	255	To detect moderate effect size, 80% power
Burst Abdomen	93	To detect moderate effect size, 80% power

The total planned cohort will consist of 500 patients, which ensures sufficient power to assess primary and secondary outcomes while allowing for exploratory and subgroup analyses, including risk factors associated with specific surgical techniques or patient characteristics. This sample size also accommodates a potential dropout rate of up to 10% and supports the development of multivariate models with adequate statistical reliability.

To ensure feasibility across all participating centres, over-recruitment will be limited to a maximum of 50% above the calculated minimum sample size required for primary endpoints.

### Authorship Criteria

Centres enrolling ≥40 patients may nominate two named authors. Centres recruiting 20–39 patients may nominate one named author. Centres enrolling fewer than 20 patients will be included in the collaborative authorship group.

## Discussion

The PROPHECY trial is uniquely positioned to provide high-quality prospective data on the incidence and risk factors for postoperative hernias in emergency abdominal surgery. This area of surgical care remains underrepresented in current literature, where most evidence derives from elective procedures, despite the fact that emergency settings present unique challenges related to patient stability, contamination, and time constraints.

Patient-related risk factors such as obesity, smoking, immunosuppression, and malnutrition are well-established contributors to impaired wound healing and hernia formation [4, 7, 11]. Yet in the emergency setting, prehabilitation and optimisation are rarely feasible. Identifying high-risk patients based on baseline characteristics may enable the development of targeted intra- and postoperative strategies.

The adoption of the “small bites” technique in fascial closure has been shown to reduce the risk of incisional hernia in elective settings [8, 9], yet its effectiveness in the context of emergency procedures remains to be confirmed. In their meta-analysis, Henriksen et al. demonstrated that small bites using slowly absorbable sutures reduced hernia risk significantly compared to larger bites [8]. However, most trials excluded contaminated or emergency cases. The PROPHECY trial will help determine whether these findings translate to emergency settings where tissue handling is often suboptimal.

Port-site hernias, while historically underestimated, may occur in up to 30% of patients after laparoscopic procedures depending on the size and location of trocar incisions [5, 12]. The decision to suture fascial defects greater than 10 mm is based on such data and is now considered best practice [9]. However, data on port-site hernias from emergency laparoscopies remain limited. The PROPHECY trial will provide new insight into this complication in urgent settings.

Parastomal hernias remain another prevalent but under-reported complication. A study by Antoniou et al. found that up to half of patients with permanent stomas develop a hernia at the stoma site [6]. Evidence supports the use of prophylactic mesh in elective stoma formation to reduce this risk [6, 13], but the safety and feasibility of mesh use in emergency stoma creation is still debated due to concerns regarding infection. A meta-analysis by Elzeftawy et al. demonstrated that mesh implantation—particularly in the retromuscular position—significantly reduces the risk of parastomal hernia without increasing the incidence of surgical site infections [13].

One of the most important modifiable factors identified in the literature is surgical site infection (SSI), which significantly increases the risk of incisional hernia development [7, 12]. The use of prophylactic negative pressure wound therapy (NPWT) on closed incisions has shown promise in reducing SSI rates, particularly in high-risk or contaminated fields [14]. Nevertheless, adoption of NPWT in emergency settings remains inconsistent. PROPHECY will capture detailed data on the use of NPWT and its association with hernia and wound-related outcomes.

The inclusion of burst abdomen as a secondary endpoint reflects the growing awareness of this severe complication. Kvist et al. recently reported that burst abdomen following emergency laparotomy occurred in 4.4% of patients and was associated with significantly higher rates of morbidity and mortality [10]. This underlines the importance of optimal fascial closure and early identification of wound dehiscence in this population.

Another area of interest is postoperative care, including the use of abdominal binders and guidance on physical activity resumption. While widely practised, there is limited high-quality evidence supporting their role in preventing hernia formation [2, 9]. PROPHECY will contribute to clarifying the effectiveness of these interventions.

Finally, the multicentre, prospective design and robust sample size calculation of the PROPHECY trial will allow for meaningful subgroup analyses and generalisable conclusions.

By identifying both patient- and procedure-related modifiable risk factors, this study has the potential to inform the creation of specific guidelines for hernia prevention in emergency surgery, where currently none exist.

## Data Availability

The original contributions presented in the study are included in the article/supplementary material, further inquiries can be directed to the corresponding author.
